# Glycosyl hydrolase 11 (*xynA*) gene with xylanase activity from thermophilic bacteria isolated from thermal springs

**DOI:** 10.1186/s12934-022-01788-3

**Published:** 2022-04-15

**Authors:** Johnson Beslin Joshi, R. Priyadharshini, Sivakumar Uthandi

**Affiliations:** 1 Department of Plant Biotechnology, Centre for Plant Molecular Biology and Biotechnology, Tamil Nadu Agricultural University, Coimbatore, 641003 India; 2 Biocatalysts Laboratory, Department of Agricultural Microbiology, Tamil Nadu Agricultural University, Coimbatore, 641 003 India

**Keywords:** Hemicellulose, Hydrolysis, Xylanase, *xynA*, Thermophiles

## Abstract

**Background:**

Hemicellulose is one of the copious polymer in lignocellulosic biomass (LCB). It is primarily composed of xylan linked by β-1,4 glycosidic bonds. Xylanase preferentially cleaves the β-1,4-glycosidic bonds in the xylan backbone resulting in complete hydrolysis of the biomass. Thermostable variants of glycoside hydrolases act as robust catalysts, not only in degradation but also during processing, to obtain specific carbohydrate-containing chemicals and materials (Ramasamy et al. in Madras Agric J 107(special):1. 10.29321/MAJ.2020.000382, 2020).

**Results:**

The xylanase production by two thermophilic bacteria isolated from thermal springs was evaluated. In addition, the gene encoding this industrially vital enzyme was isolated and characterized, and its protein structure was analyzed. The thermophilic bacteria producing xylanases were isolated from augmented sawdust and banana fiber biomass from hot springs of Himachal Pradesh and identified as *Bacillus subtilis* VSDB5 and *Bacillus licheniformis* KBFB4 using 16S rRNA gene sequencing. The persistent xylanase activity revealed that the enzyme is secreted extracellularly with the maximum activity of 0.76 IU mL^−1^ and 1.0 IU mL^−1^ at 6 h and 12 h of growth by KBFB4 and VSDB5, respectively, under submerged fermentation. Both the strains exhibited the maximum activity at pH 6 and a temperature of 50 °C. The xylanases of KBFB4 and VSDB5 were thermostable and retained 40% of their activity at 60 °C after incubation for 30 min. Xylanase of VSDB5 had wide thermotolerance and retained 20% of its activity from 60 to 80 °C, whereas xylanase of KBFB4 showed wide alkali tolerance and retained 80% of its activity until pH 10. The xylanase (*xynA*)-encoding gene (650 bp) cloned from both the strains using specific primers showed 98 to 99% homology to *β-1,4-endoxylanase* gene. Further in silico analysis predicted that the xylanase protein, with a molecular weight of 23 kDa, had a high pI (9.44–9.65), which explained the alkaline nature of the enzyme and greater aliphatic index (56.29). This finding suggested that the protein is thermostable. Multiple sequence alignment and homology modeling of the protein sequence revealed that the gene product belonged to the GH11 family, indicating its possible application in bioconversion.

**Conclusion:**

The strains *B. subtilis* VSDB5 and *B. licheniformis* KBFB4 obtained from hot springs of Himachal Pradesh produced potent and alkali-tolerant thermostable xylanases, which belong to the GH11 family. The enzyme can be supplemented in industrial applications for biomass conversion at high temperatures and pH (or in processes involving alkali treatment).

**Graphical Abstract:**

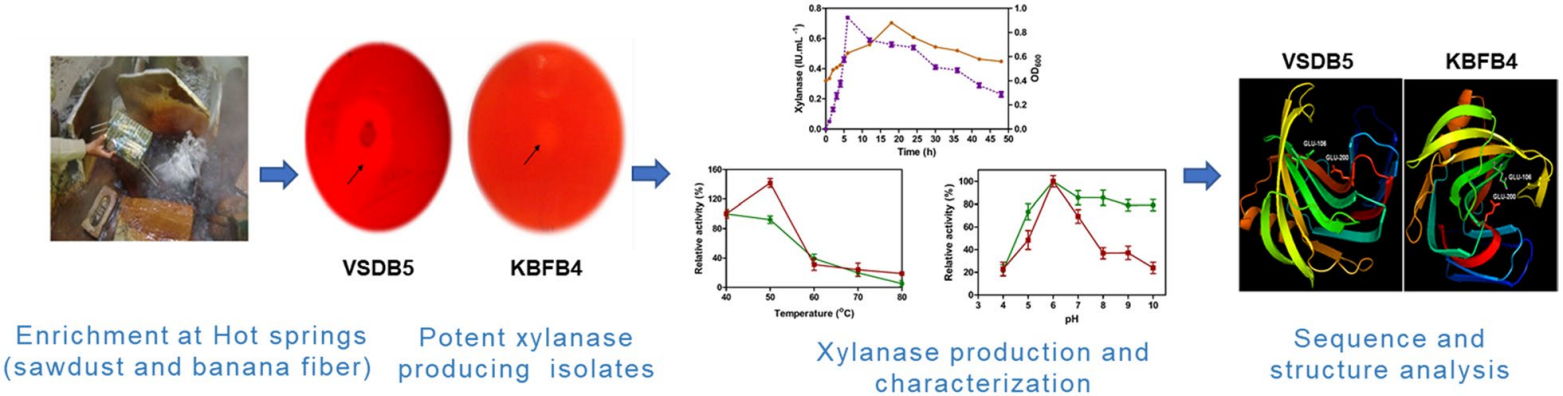

**Supplementary Information:**

The online version contains supplementary material available at 10.1186/s12934-022-01788-3.

## Background

Hemicelluloses, one of the abundant polymers in nature, represent about 20 to 35% of lignocellulosic biomass, among which xylan is the most abundant. In recent years, the bioconversion of xylan has received considerable attention because of its practical applications in several agro-industrial processes, such as the competent conversion of hemicellulosic biomass to fuels and chemicals, delignification of paper pulp, digestibility enhancement of animal feedstock, clarification of juices, and upgrading the consistency of beer [1, 2]. Xylanase (endo-1,4-β-xylanase, EC 3.2.1.8) is a hydrolytic enzyme that arbitrarily cleaves the β-1,4 backbone of the complex plant cell wall polysaccharide, xylan. In addition, endo-β-1,3-xylanases (EC.3.2.1.32) from seaweed that targets β-1,3-linked xylose polymer have also been described. In glycosyl hydrolase (GH) families 3, 5, 8, 9, 10, 11, 12, 16, 26, 30, 43, 44, 51, 62, 98, and 141, the endo-β-1,4-xylanases are found, whereas GH families 11 and 26 holds endo-β-1,3-xylanases as reported in CAZy database [3, 4]. Xylanases from GH families 5, 7, 8, 10, 11, and 43 have a single distinct catalytic domain, whereas GH families 16, 51, and 62 had two catalytic domains with bi-functional property [3]. More scientific insights on the mechanism of action of xylanase were focused on the glycosyl hydrolase family 10 and 11. The members of the GH11 family (also known as true xylanases) xylanases are more specific to unsubstituted xylan substrate, while GH10 hydrolyze substituted xylan backbone. Xylanase hydrolysis is mediated through the classical Koshland double-displacement mechanism, in which each substrate contributes hydroxyl groups to the stabilization of the transition state, leading to catalysis. The xylanase sequence is composed of one or more non-catalytic carbohydrate-binding modules (CBMs) for xylan recognition and catalytic domain for hydrolysis. The three-dimensional structures of GH10 and GH11 xylanase have distinct (α/β)8 barrel fold and β-jelly roll fold structures, respectively, with glutamate and aspartate residues in the catalytic site [2, 3].

Although xylanases have been reported from diverse organisms, thermophilic xylanase producers have gained significance due to their ability to operate under industrial process conditions. Further, the exploration of novel thermophilic biocatalysts for xylan conversion could be economically beneficial. Such enzymes are involved in the saccharification of several pretreated agricultural and forest residues to fermentable sugars and imitative products like xylitol, ethanol, furfural, and various functional biopolymers [5, 6]. The exploration of hot springs for thermophiles producing biomass-degrading enzymes led to the identification of biocatalysts with multi-functional glycosyl hydrolases [7]. Previously, the thermophilic bacterium *Bacillus aerius* CMCPS1, isolated from the thermal springs of Manikaran, Himachal Pradesh, India, was characterized for its cellulase activity, stability, and hydrolytic capacity. It exhibited enhanced saccharification of corn cob biomass [8]. Hence, the isolation of the xylanase gene and its co/over-expression with other biomass-degrading enzymes as part of a bi/multi-functional system will further amplify the process efficiency. With this background, the present research was focused on isolating xylan-degrading thermophilic bacteria from the hot springs of Himachal Pradesh. The full-length xylanase gene was cloned, and its sequence and structure were predicted. In addition, we conducted in silico physicochemical analysis and delineated its enzyme production potential.

## Results

### Isolation of xylanolytic thermophilic bacteria from hot springs of Himachal Pradesh

Two thermophilic bacteria, namely VSDB5 and KBFB4, were isolated from in-situ enriched sawdust and banana fiber biomass samples (Additional file 1: Fig. S1a), respectively, from the hot springs of Vashisht (~ 65 °C) and Kalath (~ 65 °C), Himachal Pradesh. The xylan-degrading ability of these strains was assessed qualitatively by substrate hydrolysis and visualized by the formation of a clear yellow zone around the positive colonies (Additional file 1: Fig. S1b). VSDB5 and KBFB4 exhibited a hydrolytic capacity of 5.5 and 1.35, respectively, at 50 °C. Colonies of KBFB4 appeared straw yellow and had granular consistency, and those of VSDB5 appeared off-white and had creamy consistency on tryptic soy agar (Additional file 1: Fig. S1c).

### Identification of bacteria

**Fig. 1:**
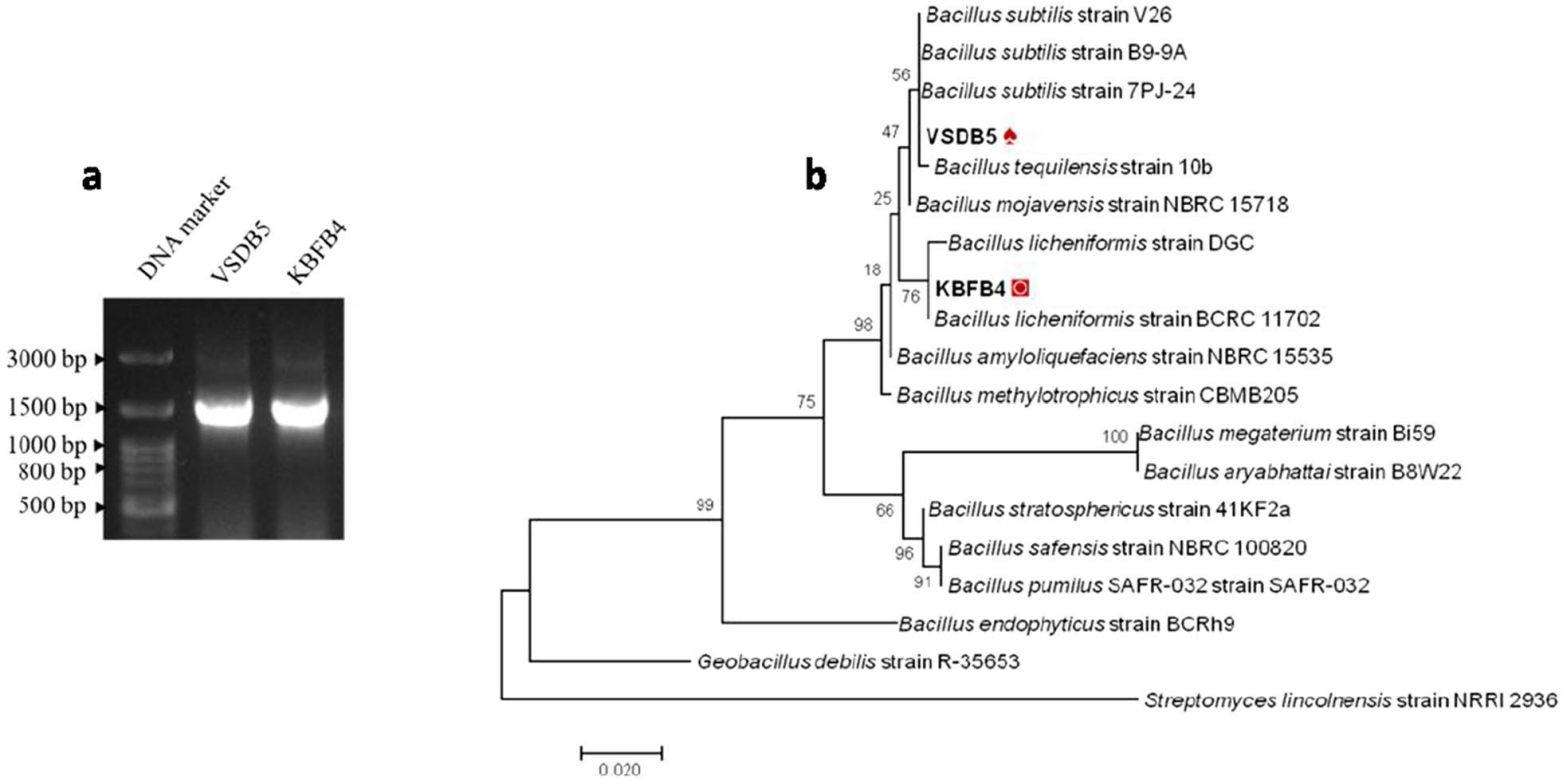
Molecular identification of thermophilic bacteria. **a** PCR amplification of 16S rRNA gene from thermophilic bacterial isolates. **b** Neighbor-joining phylogenetic tree constructed based on the 16S rRNA gene sequences of thermophilic bacteria. The number at branches indicates bootstraps value (> 50%) from 1000 replicates

Potential xylanolytic thermophilic isolates, VSDB5 and KBFB4, were analyzed phylogenetically using 16S rRNA gene sequencing (Fig. 1a), which showed 99% homology to *Bacillus subtilis* strain 7P J-24 (KR708834.1) and *B. licheniformis* strain MA1B (KX270723.1), respectively. The 16S rRNA gene sequences of VSDB5 and KBFB4 were deposited in the NCBI GenBank under the accession numbers MF187473.1 and MF187475.1, respectively. The strains belong to the phylum Firmicutes. A phylogenetic tree was constructed based on the aligned datasets with MEGA 6.0 using the neighbor-joining (NJ) method (Fig. 1b).

### Growth and xylanase production

**Fig. 2:**
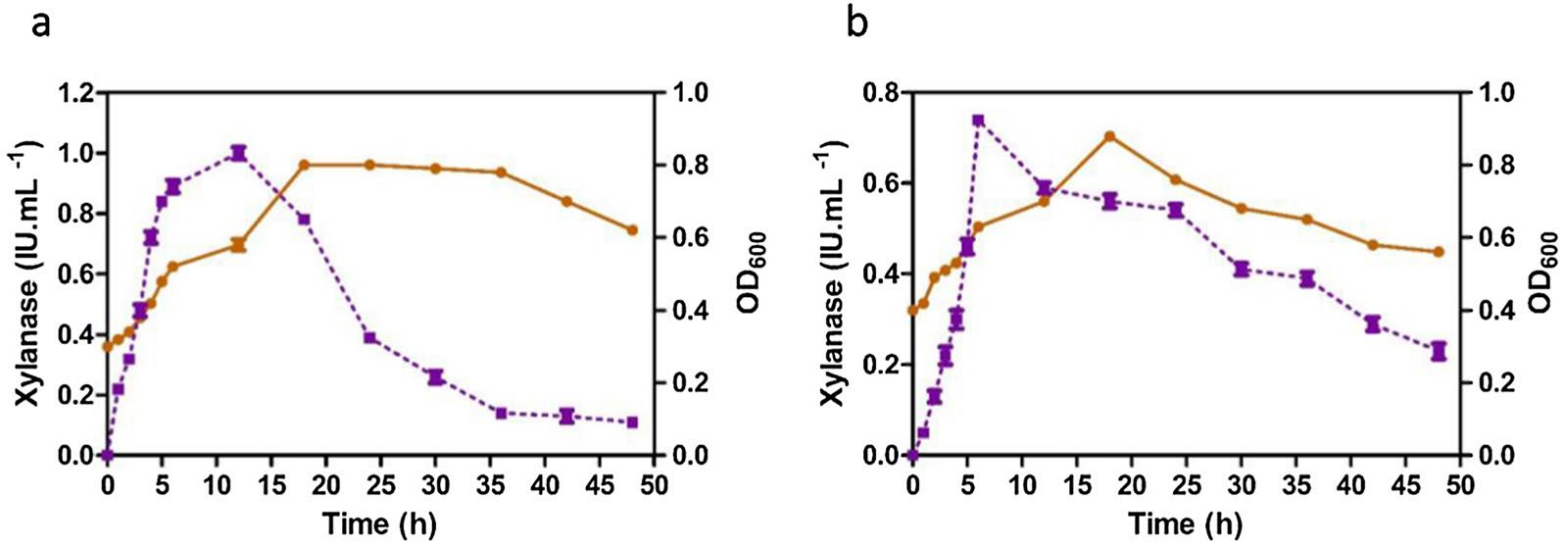
Growth and xylanase production in thermophilic bacteria VSDB5 (**a**) and KBFB4 (**b**). Growth is expressed as a measure of OD at 600 nm, depicted as a continuous line (orange), and xylanase activity in IU mL^−1^ is depicted as dotted lines (purple). The maximum activities for KBFB4 and VSDB5 were 0.76 and 1.0 IU mL^−1^ at 6 and 12 h after inoculation, respectively. The standard deviation was not more than 5% from the mean

The growth of xylanolytic strains in the xylan-containing minimal medium increased gradually until 6 h after inoculation, reaching the maximum activity at 18 h. VSDB5 exhibited the maximum growth of the two strains with an optical density of 0.8 at 18 h. In addition, the xylanase activity increased with increased bacterial growth. The maximum activities of 0.76 and 1.0 IU mL^−1^ were observed at 6 and 12 h after inoculation for KBFB4 and VSDB5, respectively (Fig. 2a, b). After 36 h, the xylanase activity reduced by 50% in both the strains, which could be due to the effect of proteases produced by *Bacillus* spp. (Fig. 2a, b).

### Effect of pH on xylanase activity and stability

**Fig. 3:**
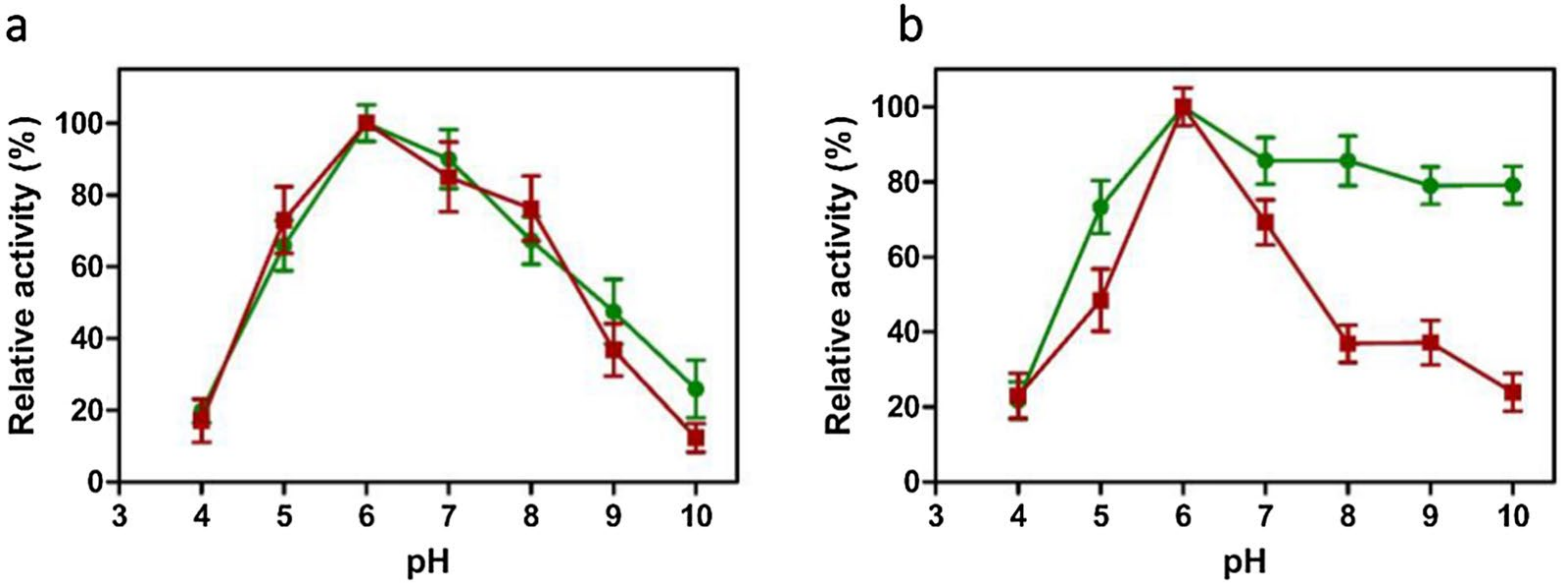
Effect of pH on the activity (**a**) and stability (**b**) of xylanase from KBFB4 and VSDB5. The optimal pH for xylanase activity was measured at 50 °C for 30 min in different buffer systems, namely, citrate phosphate (100 mM, pH 3 to 8) and glycine (100 mM, pH 8 to 10). The pH stability was determined by incubating the enzymes at various pH values for 30 min, followed by enzyme assay. The maximum activity was expressed as the activity maxima 100%. The KBFB4 and VSDB5 are represented by filled circles (green) and filled squares (red), respectively. The standard deviation was not more than 10% from the mean

The enzyme displayed a wide pH activity profile ranging from pH 5 to 8. The maximum xylanase activity was observed at pH 6 for both strains. In both VSDB5 and KBFB4 strains, the relative activity of xylanase did not vary considerably across different pH values (4 to 10). Xylanases from both the strains retained more than 80% of their activity at pH 7 and more than 60% of their activity at pH 5 and 8 (Fig. 3a). The stability of KBFB4 xylanase at different pH values indicated that KBFB4 was more alkali stable than VSDB5 and retained about 80% of its activity at a wide range of pH from 7 to 10. The xylanase from VSDB5 depicted a sharp decrease in the relative activity from pH 6 to 8, retaining 65% of its relative activity at pH 7 and 38% activity at pH 8 to 9. Finally, at pH 10, only 20% of the xylanase activity was retained by the strain VSDB5 (Fig. 3b).

### Effect of temperature on xylanase activity and stability

**Fig. 4:**
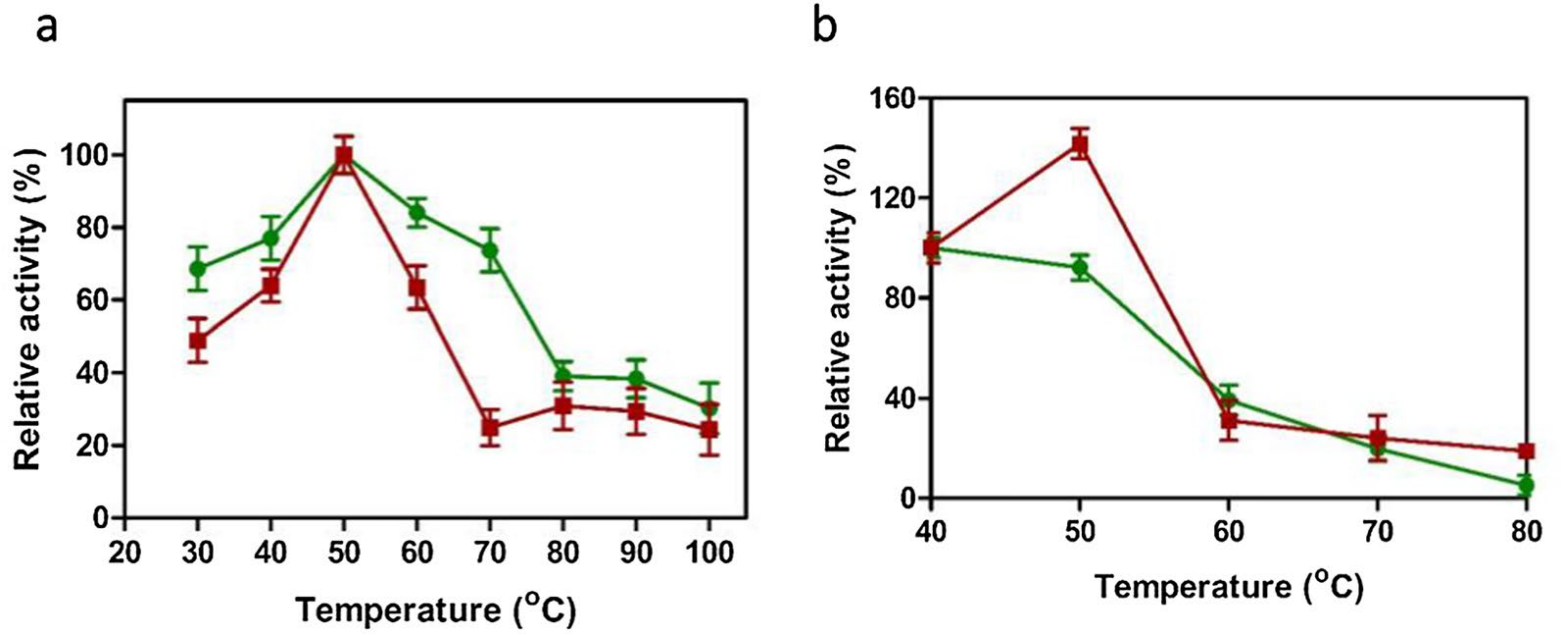
Effect of temperature on the activity (**a**) and stability (**b**) of xylanase from KBFB4 and VSDB5. The optimal activity was observed at 50 °C was expressed as the activity maxima 100% (**a**) and the activity at 40 °C was expressed as the activity maxima 100% (**b**). The optimal temperature for xylanase activity was measured at different temperatures ranging from 30 to 100 °C (**a**). To determine the thermostability, the enzyme was incubated at 40 °C, 50 °C, 60 °C, 70 °C and 80 °C for 30 min, followed by xylanase assay under standard conditions at pH 5 and 50 °C (**b**). The KBFB4 and VSDB5 are represented by filled circles (green) and filled squares (red), respectively. The deviation was not more than 10% from the mean

The optimum temperature for the xylanase activity of VSDB5 and KBFB4 was found to be at 50 °C. The VSDB5 and KBFB4 enzymes had 63% and 84% of their activities at 60 °C, respectively. At 70 °C, VSDB5 retained only 25% of its activity, whereas KBFB4 exhibited 73% of its activity. The xylanase activity was reduced after 80 °C for VSDB5 and 90 °C for KBFB4 (Fig. 4a).

The temperature stability of the xylanase of VSDB5 and KBFB4 was tested at pH 5 by incubating the enzyme preparations at 40, 50, 60, and 70 °C for 30 min. The enzyme was highly stable; the KBFB4 xylanase retained about 95% of its activity at 50 °C, whereas that of VSDB5 had a 40% increase. However, the relative enzyme activity of VSDB5 and KBFB4 was reduced to 40% of their initial activity at 60 °C. A 10-degree increase in the temperature greatly affected the enzyme stability, resulting in the loss of xylanase activity to half after 30 min of incubation. Xylanase from VSDB5 retained 20% of its activity at a wide range of temperature from 60 to 80 °C, exhibiting stable tolerance. The relative activity of xylanase produced by KBFB4 declined gradually from 60 to 70 °C, ultimately reaching its minimum at 80 °C after 30 min of incubation time (Fig. 4b).

### Isolation of xylanase gene

**Fig. 5:**
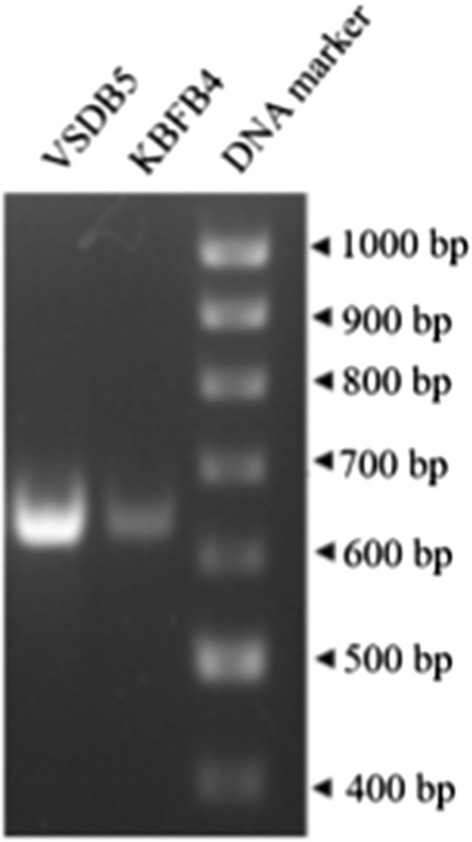
PCR amplification of xylanase gene in thermophilic bacteria. Xylanase gene-specific PCR resulted in the amplification of a gene product of around 650 bp in both isolates

The xylanase gene (650 bp) from the thermophilic bacteria VSDB5 and KBFB4 was isolated using xylanase-specific primers (Fig. 5). The sequence of the purified xylanase gene fragment was analyzed using NCBI BLAST, and the closest match from the GenBank data was reported (Table 1). The full-length xylanase gene sequence obtained in this study from *B. subtilis* VSDB5 and *B. licheniformis* KBFB4 was submitted in the NCBI database under the Accession numbers MF288580 and MF288581, respectively.

**Table 1:** Analysis of gene sequence of xylanase from *Bacillus* sp. strains VSDB5 and KBFB4

Primer used	Sample	Sequence homology			
		Organism	Gene	Accession no	Percent homology^a^
Xylanase	VSDB5	*Bacillus subtilis *subsp. *subtilis* strain *D12-5*	*1,4-beta-xylanase (GH11)*	CP014858.1	99
	KBFB4	*Bacillus licheniformis* isolate *MS5-14*	*β-1,4-endoxylanase* (*Xyn11*)	EU591524.1	98

^a^Values presented are the percentage of nucleotide sequence homology between VSDB5 and KBFB4

### Protein modeling

**Fig. 6:**
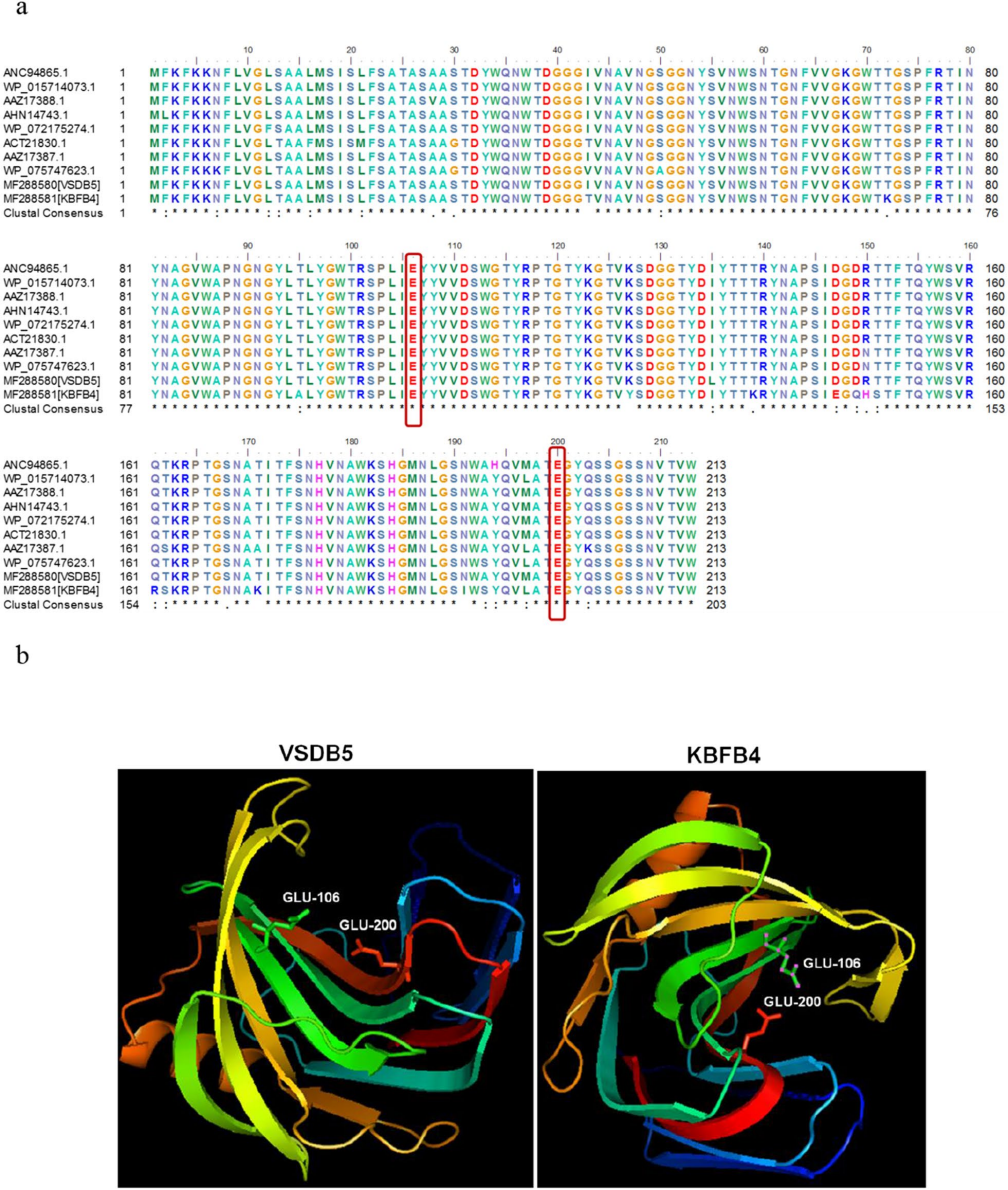
Multiple sequence alignment of xylanase protein sequence (**a**) and its predicted structure (**b**). Xylanase-encoding protein sequences retrieved from NCBI were aligned with xylanase sequences of thermophilic isolates using BioEdit sequence editor (Version 7.2.5). The consensus symbols ‘*’ means residues are identical, ‘:’ means conserved substitutions, ‘.’ means semi-conserved substitutions in the alignment. The active site region is shown in blue boxes. The structure was predicted in the Swiss-Homology Modeling server and the active sites are indicated

The xylanase gene (642 bp) codes for 213 amino acids, and the multiple sequence alignment of the xynA (GH11) protein sequence showed 98 to 99% homology with β-1,4-endoxylanase from other *Bacillus* spp. (Fig. 6a). The identity and similarity between the xylanase sequence from the strains VSDB5 and KBFB4 were 91.5% and 95.8%, respectively. Multiple sequence alignment of the amino acid sequence showed the presence of positively charged amino acids lysine (Lys) at K72, K139, K171 positions and arginine (Arg) at R161 position in KBFB4 xylanase, while other xylanase proteins showed no charged amino acids, threonine (Thr) at T72, T139, and T171, and glutamine (Gln) at Q161 position, respectively. Furthermore, the presence of lysine (Lys), histidine (His), serine (Ser), and tyrosine (Tyr) at K72, K139, K171, H150, S151, S162, and Y127 that might be involved in hydrogen bonding, and a salt bridge formation was noticed in KBFB4 (Fig. 6a). In addition, homology modeling of xylanase protein from strains VSDB5 and KBFB4 using 2dcy.1.A and 1xxn.1.A as templates, respectively, in a SWISS homology modeling server, revealed that the two glutamic acid residues forming the active site were at positions 106 and 200 for both xylanases (Fig. 6b), and the positions significantly different amino acids are depicted in the structure of VSDB5 and KBFB4 xylanase (Fig. 6c). QMEAN values of xylanase protein from VSDB5 and KBFB4 were found to be 0.91 and 0.90, respectively. Structure analysis MolProbity results revealed 96.70% and 96.15% of the amino acids in VSDB5 and KBFB4 were in the favorable region. The secondary structure analysis of VSDB5 and KBFB4 strains revealed an α-helix mean value of 20.19 and 14.55%, beta-turn region of 7.98 and 7.02%, extended sheet region of 33.8 and 34.27%, and a random coil of 38.03 and 44.13%, respectively. The secondary structure analysis revealed a structure consisting of random coils, extended sheets, α-helices, and to a lesser extent, beta-turn regions (Additional file 1: Fig. S2).

### In silico analysis of physicochemical parameters of xylanase

Physical parameters of thermophilic bacterial xylanase were analyzed in silico by studying the physicochemical parameters*,* namely the number of amino acids and composition, molecular weight, theoretical pI, extinction coefficient, instability index, aliphatic index, grand average of hydrophobicity (GRAVY) using the ExPASy-ProtParam tool. The results are summarized in Table 2. The predicted molecular weight of xylanase was 23 kDa for VSDB5 and KBFB4. Theoretically, the pI value of more than 7 represented the alkaline nature of the protein. The xylanase protein recorded a high aliphatic index of 56.29 and a negative GRAVY value (grand average of hydrophobicity). The instability index indicated that the protein is highly stable. Glycine, threonine, and serine were the predominant amino acids in both the xylanases with the signal peptide cleavage site at 28 and 29 amino acid positions (Table 2).

**Table 2:** Physicochemical parameters predicted for xylanase proteins from *Bacillus* sp. strains VSDB5 and KBFB4

Parameters	VSDB5	KBFB4
Molecular weight (Da)	23,358.71	23,478.94
Theoretical pI	9.44	9.65
Extinction coefficient (M^–1^ cm^–1^)	82,850	84,340
Instability index	15.87	23.66
Aliphatic index	56.29	56.29
Grand average of hydrophobicity (GRAVY)	− 0.448	− 0.448
Signal peptide cleavage site (Sec/SPI) amino acid position	28 and 29	28 and 29
Predominant amino acids (Glycine, Threonine, and Serine) (%)	12.2, 12.2, and 10.8	12.2, 10.3, and 11.3

## Discussion

Xylanase, an industrially important enzyme, is one of the most widely studied groups of hemicellulases from bacteria and fungi, owing to their several biotechnological applications [9]. Although xylanases from microorganisms have been characterized in detail and are commonly used in pulp and paper processes, there is an increased need for a thermostable enzyme for industrial use. Such enzymes can endure high temperatures and have improved robustness, in addition to increased reaction rate, which eventually decreases the amount of enzyme required for bioprocessing [10]. Consequently, the exploration of novel biocatalysts from thermal springs is of great importance, owing to their intriguing biogeochemistry and adaptations to extreme environments. Moreover, thermophilic microorganisms have gained importance worldwide due to their tremendous potential to produce thermostable enzymes with broad applications in various industries. Studies have been conducted to enrich hemicellulose-abundant lignocellulosic biomass such as sawdust [11] and banana fiber [12] that favor the growth of hemicellulose-degrading bacteria under thermophilic conditions to about 15 to 18%, and 14.98%, respectively. Similarly, Eichorst et al. [13] isolated cellulose-and hemicellulose-degrading bacteria following the substrate-specific enrichment technique. In this regard, we isolated xylanase-producing thermophilic bacteria from thermal springs of Himachal Pradesh and evaluated their genes and enzyme production potential.

Thermophiles isolated from the hot spring by in-situ enriched sawdust and banana fiber biomass samples efficiently hydrolyzed the xylan substrate. The majority of xylanase-producing isolates were identified based on 16S rRNA sequencing as *Bacillus subtilis* VSDB5 and *B. licheniformis* KBFB4, belonging to the phylum Firmicutes (Fig. 1b). Several bacterial genera, such as *Bacillus*, *Cellulomonas*, *Micrococcus*, *Staphylococcus*, *Paenibacillus*, *Arthrobacter*, *Microbacterium*, *Pseudoxanthomonas*, and *Rhodothermus* have been reported to produce xylanases [14–17]. Among bacteria, *Bacillus* spp. are an important source of xylanases, and several *Bacilli,* such as *B. circulans, B. stearothermophilus, B. amyloliquefaciens, B. subtilis, B. pumilus,* and *B. halodurans* [18–21], have been reported to have considerable xylanolytic activity.

KBFB4 and VSDB5 had the maximum xylanase activities of 0.76 and 1.0 IU mL^−1^ at 6 and 12 h, respectively, after inoculation. The xylanase production increased with an increase in bacterial growth (Fig. 2a, b). A similar trend in bacterial growth and xylanase activity was also reported previously [22]. In contrast to other extracellular enzymes, β-xylanases are constitutively expressed and synthesized mostly during the exponential growth phase [20]. In the present study, a decrease in xylanase production to 50% was observed after 36 h of fermentation, probably due to the effect of proteases produced by *Bacillus* spp. This decrease in endo-1,4-β-xylanase production could also be due to the feedback inhibition caused by the accumulation of the end product (xylose) during the hydrolysis of xylan [23]. Furthermore, the depletion of macro and micronutrients in the growth medium and the shift in medium pH might also be responsible for a declined bacterial growth and enzyme production [24]. Xylanase production by *B. subtilis* increased from 12 to 96 h with wheat bran as a substrate (contains 20% to 40% of hemicelluloses) [24]. However, we observed that VSDB5 and KBFB4 produced a high yield of endo-1, 4-β-xylanase within a short period.

Several reports on pH and temperature optima studies from *Bacillus* spp. are available. Both VSDB5 and KBFB4 showed optimal activity at 50 °C and pH 6.0 (Figs. 3a, 4a) with moderate thermostability at 50 °C. The xylanases from *B. subtilis* had optimal activity at pH 6.0 and 50 °C [25]; the Indonesian *B. licheniformis* exhibited optimal activity at pH 7 and 50 °C. In addition, the recombinant xylanase from *B. licheniformis* strain I5 had optimal activity at pH 7 and temperature stability at 50 °C [26]. The thermostability of the recombinant xylanase was determined at 40, 50, and 55 °C. The enzyme retained more than 90% of its activity after 30 min incubation at 40 °C; however, the residual activity decreased at 50 and 55 °C. The residual activity after 10 min of incubation at 50 °C was 60%, and no activity was recorded at 55 °C [26]. *B. subtilis* M015 XynA displayed optimum activity at pH 5.5 to 6.0 and temperature of 50 °C and retained over 50% relative activity between 37 and 60 °C [27]. The *B. licheniformis* Xyn11 enzyme had an optimal activity at pH 5 to 7 and temperatures of 40 to 50 °C [28].

Among the members characterized so far, the optimal temperature range for xylanase has been reported to range from 35 to 85 °C. However, the optimal temperature range of thermophilic Xyl-11 is from 62 to 85 °C [29]. A thermostable Xyl-11 with optimal temperatures from 70 to 80 °C has been reported from *Bacillus* sp. JB-99 [30]. In our study, the strains KBFB4 and VSDB5 had optimum pH of 6 and temperature of 50 °C, which is consistent with the earlier reports. The thermostability was higher than that reported for xylanases isolated from *B. subtilis* M015 and *B. licheniformis* Xyn11. The xylanase from VSDB5 and KBFB4 identified in this study retained 63% and 84% of its activity at 50 °C and 25% and 73% at 60 °C. Furthermore, the *B. licheniformis* KBFB4 retained about 40% of its activity even at high temperatures ranging from 80 to 90 °C (Fig. 4b). This wide range of xylanase activity from 50 to 70 °C can be efficiently exploited for industrial operation. Although a wide range of enzyme activity across different temperatures was observed in *B. licheniformis* KBFB4, the *B. subtilis* VSDB5 had greater thermostability. The alkali-tolerant xylanases are vital in the biomass conversion process for higher sugar recovery and biofuel production. Earlier studies reported that mesophilic *Bacillus* spp. MX47 was grown at 40 °C for alkaliphilic xylanase production [31]. *B. licheniformis* KBFB4 xylanase was alkali tolerant and retained around 80% of its activity at a higher pH of 7.0 to 10.0 (Fig. 3b). Such positive attributes of short fermentation time (12 h) and xylanase production at moderately higher temperatures with alkali tolerance revealed that *B. subtilis* VSDB5 and *B. licheniformis* KBFB4 could be efficient candidates in different industrial processes. The isolation and sequencing of the xylanase gene (650 bp) indicated that the strains produced the GH11 xylanase (Fig. 5). The whole-genome sequencing of *B. subtilis* 168 revealed that the gene *xynA* (1241 bp), encoding endo-1,4-β-xylanase (GH11 family) enzyme, is located on chromosomal DNA [9]. Previously, the xylanase gene *xynA* (GH11 family) was isolated from several organisms such as *Bacillus circulans*, *Bacillus subtilis, Streptomyces* sp. S38, *Thermopolyspora flexuosa*, *Escherichia coli,* and *Thermobifida fusca* [9] and cloned into suitable hosts for overexpression [32]. Previously, two xylanase genes (*xynA* and *xynB*) from *Paenibacillus* sp. KCTC 8848P and *Ruminococcus flavefaciens* 17 were cloned and expressed in a heterologous host *E. coli* [33, 34]. Similarly, a xylanase gene of 642 bp length was cloned from *B. subtilis* B10 and expressed in *E. coli*. The distribution of xylanase in extracellular, intracellular, and periplasmic fractions is about 22.4, 28.0, and 49.6%, respectively [25].

The sequence-structure analysis of xylanase from thermophilic *B. subtilis* VSDB5 and *B. licheniformis* KBFB4 (Fig. 6a, b) revealed a xylanase structure similar to that of other xylanases from the GH11 family, the presence of one α-helix and two strongly twisted *β*-sheets forming a cleft on one side of the protein and consisting of the active site displayed a β-jelly roll architecture [35]. The predicted molecular weight of the xylanase protein was 23 kDa, with an alkaline pI, explaining the alkaline nature of the xylanase. Furthermore, the presence of basic amino acids, lysine (K72, K139, K171), arginine (R161), and histidine (H150) in the KBFB4 sequence can favor the xylanase activity at alkaline pH. Engineering positively charged arginine residues onto the xylanase protein surface improved the enzyme adaptation to highly alkaline pH levels [36]. Recently, Wu and his co-workers observed excess of positively charged residues on the surface of *BsXynJ*, an alkali-stable xylanase protein from *B. subtilis,* and reported that changes in the negatively and positively charged surface amino acid ratio might contribute to their extreme pH adaptation [37]. Enzyme thermostability can be increased by promoting optimal interactions such as improved packing of the protein, hydrophobic and electrostatic interactions between the amino acids in the peptide chain [38]. In addition, hydrophobic residues are significantly more frequent in thermophilic proteins, while polar residues are less, and thermostability is achieved through few structural modifications even with few amino acids differences [39]. The presence of hydrophobic tyrosine at Y127 and uncharged glutamine at Q149 might favor the enzyme stability; in contrast, VSDB5 xylanase contains charged aspartic acid. Among the different protein sequences compared, KBFB4 showed less frequency of threonine residue (Fig. 6a). In general thermophilic enzymes have a lower prevalence of uncharged polar amino acids, mainly threonine, serine, cysteine, and glutamine, compared to the mesophiles [40–43].

Further, the replacement of threonine to lysine residue at K72, K139, K171 position might stabilize the enzyme through electrostatic interaction, explaining the enzyme stability of thermophiles [44]. The alignment of 60 protein sequences from thermophilic and their mesophilic homologs revealed a replacement of 25% amino acids with charged ones in thermophiles [39]. A high aliphatic index of 56.29 suggested the high thermostability of the protein. A negative GRAVY value indicated that the protein is non-polar in nature (Table 2). From the metagenomic DNA of compost soil, the gene for an alkaline tolerant and thermostable xylanase (*Mxyl,* 1077 bp) encoding 358 amino acids (GH 11 family) was cloned into the pET28a vector and expressed in *E. coli* BL21 (DE3). The recombinant xylanase (rMxyl) exhibited activity over a broad range of pH and temperature, with the optimum activity at pH 9.0 and 80 °C [45, 46]. Bilgin et al. [47] demonstrated that the *B. subtilis* xylanase (26 kDa) expressed in *E.coli* had an optimal activity at pH 6.0 and 60 °C. The signal peptide cleavage site was found in the protein sequence at residue 28, 29 amino acids confirmed the secretion of xylanase enzyme into the extracellular medium (Table 2). The thermophilic alkaline-tolerant xylanase secreted extracellularly by *B. subtilis* VSDB5 and *B. licheniformis* KBFB4 can be efficiently purified and used in several industrial processes. Furthermore, xylanases with several industrial applications have been produced at a large scale from diverse fungal sources [48]. There will be a huge demand for thermostable xylanases in the future because of their role in biorefineries [49]. Hence, the xylanase gene isolated from thermophilic bacteria in the present study can be massively articulated for commercial xylanase production.

## Conclusion

The conversion of xylan into useful value-added products by enzymatic and fermentation route holds a great promise for various under-utilized agricultural dregs, leading to the bio-based economy. In the present study, two xylan-degrading thermophilic bacteria *Bacillus subtilis* VSDB5 and *B. licheniformis* KBFB4 isolated from biomass samples produced xylanases within 12 h of fermentation. The presence of xylanase gene fragments implies that these strains are required for xylan-derived bio-products. Xylanases of KBFB4 and VSDB5 were thermostable and retained 40% of their activity at 60 °C with a wide pH activity of 6 to 8. Higher pI and aliphatic index explained the thermostability and alkali tolerance displayed by the enzyme. The information on gene sequence and structure gained from the present study can be used for manipulation and co-or over-expression of xylanases with other biomass-degrading enzymes to augment the efficiency of bioconversion process as well as to obtain xylan-derived products.

## Methods

### Materials

The chemicals used in the study were of analytical reagent grade or molecular biology grade obtained from Hi-media, E-Merck, Sigma Chemicals, and Bangalore Genei. The Molecular biology kits, enzymes, primers, and buffers used in the molecular analysis were obtained from M/S Thermo Scientific (USA); Biolabs (New England); Bio-Rad (India), and M/S. Sigma Aldrich (USA), respectively.

### Isolation and screening of thermophilic bacteria

The thermophilic bacteria (VSDB5 and KBFB4) were isolated from in-situ enriched sawdust and banana fiber biomass samples from hot springs of Vashisht (32.2657° N; 77.1877° E) and Kalath (32.1856° N; 77.1856° E), Himachal Pradesh, by serial dilution and plating technique. The temperatures of the Vashisht and Kalath hot springs were 42–52 °C and 39–43 °C, respectively; the water was alkaline and rich in minerals. Thermophilic xylanase producing bacteria were isolated by dilution plate on minimal agar plates (in g L^−1^: KH_2_PO_4_, 2.5; K_2_HPO_4_, 2.5; (NH_4_)_2_HPO_4_, 1.0; MgSO_4_, 0.2; CaCl_2_, 0.1; FeSO_4_, 0.01; MnSO_4_, 0.007; Agar, 20; pH 7.0) containing 1% birchwood xylan. The agar plates were incubated at 50 °C for 24 h and constantly observed for the appearance of distinct bacterial colonies.

The purified bacterial isolates were spot inoculated in 1% birchwood xylan-containing minimal media (in g L^−1^: KH_2_PO_4_, 2.5; K_2_HPO_4_, 2.5; (NH_4_)_2_HPO_4_, 1.0; MgSO_4_, 0.2; CaCl_2_, 0.1; FeSO_4_, 0.01; MnSO_4_, 0.007; Agar, 20; pH 7.0) and incubated at 50 °C for 48 h to screen for the xylanase activity. The plates were stained with 1% Congo red, followed by destaining with 1 M NaCl for 20 min each [50]. A zone of clearance around the colony suggested a xylanolytic activity. The ratio between the diameter of the clear zone to the diameter of the colony was measured as the hydrolytic capacity of the bacterial isolate.

### Growth and xylanase activity

Overnight grown bacterial cultures with OD_600_ of 0.6 were added to an Erlenmeyer flask containing minimal medium (30 mL) with 1% birchwood xylan as the substrate. The culture was inoculated at 50 °C in an orbital shaker at 110 rpm until a final OD of 0.03 was reached. Xylanase activity was determined periodically at an hourly interval for 6 h, followed by every 6 h interval until 48 h [51]. About 0.08 mL of birchwood xylan in 100 mM citrate buffer (pH 5) was incubated with 0.02 mL of the sample at 50 °C for 30 min. After incubation, 0.18 mL of dinitrosalicylic acid (DNS) was added and boiled for 5 min. This colored reaction mixture (0.04 mL) was diluted in 0.2 mL of water before measuring the absorbance at 540 nm in a multimode microplate reader (Molecular Devices, USA). One unit of xylanase activity was expressed as µmole of reducing sugars (xylose equivalent) released per min under assay conditions. Simultaneously, the growth of the bacterial isolates was recorded at 600 nm.

### Effect of pH and temperature on xylanase activity

To determine the optimal pH of xylanase activity, xylan hydrolysis was measured at 50 °C for 30 min in different buffers: citrate phosphate (100 mM, pH 3 to 8), glycine (100 mM, pH 8 to 10). The pH stability was determined by incubating the enzymes at various pH values for 30 min, followed by the standard xylanase assay as described above. To determine the optimal temperature of xylanase activity, xylan hydrolysis at pH 5 was measured at different temperatures ranging from 30 to 100 °C. To determine the thermostability of xylanase, the enzyme was incubated at temperatures ranging from 40 to 80 °C for 30 min, followed by xylanase assay under standard conditions at pH 5 and 50 °C.

### Extraction of genomic DNA

The genomic DNA from thermophilic bacterial isolates VSDB5 and KBFB4 was extracted using the CTAB method [52] and resolved on 0.8% agarose gel. The quantity and purity of the DNA were further examined using NanoDrop 2000 spectrophotometer (Thermo Scientific, USA).

### Identification of bacterial isolates

The 16S rRNA sequence analysis was performed for the molecular identification of thermophilic bacterial isolates. The PCR amplification of 16S rRNA gene was performed using universal primers 27F (5ʹ-AGAGTTTGATCMTGGCTCAG-3ʹ) and 1492R (5ʹ-ACGGCTACCTTGTTACGACTT-3ʹ) [53] with the following PCR conditions: 95 °C for 5 min; 30 cycles of 94 °C for 1 min, 55 °C for 1 min, and 72 °C for 1 min; and 72 °C for 10 min. A PCR product of 1.5 kb size was resolved by electrophoresis on 1.2% agarose gel and documented using a Gel DocXR + system (Bio-Rad Hercules, CA, USA). The amplified PCR products were purified using the GeneJET PCR Purification Kit (Thermo Scientific, USA) and sequenced at Eurofins, India. The phylogenetic tree was constructed using the associated dataset with MEGA 6.0 software and the neighbor-joining (NJ) method [54].

### Detection of the xylanase gene

Xylanase gene (*xynA*) was amplified from the genomic DNA of the thermophilic bacterial isolates, KBFB4 and VSDB5, using xylanase gene-specific primers Ba_Xln F (5ʹ ATGTTTAAGTTTAAAAAGAATTTC 3ʹ) and Ba_Xln R (5ʹ TTACCACACTGTTACGTTAG 3ʹ) [55], with the following PCR conditions: 95 °C for 5 min; 30 cycles of 94 °C for 1 min, 50 °C for 1 min, and 72 °C for 1 min; and 72 °C for 10 min. The PCR product was resolved by electrophoresis on 1.2% agarose gel and documented using a Gel DocXR + system (Bio-Rad Hercules, CA, USA). The amplified PCR products were purified using the GeneJET PCR Purification Kit (Thermo Scientific, USA) and sequenced at Eurofins, India. The acquired nucleotide sequences were analyzed for sequence similarity using BLASTn with the NCBI database.

### Sequence-structure analysis by homology modeling

The translated amino acid sequence of the full-length xylanase gene sequence was used in the sequence-structure analysis. Protein sequences of xylanases obtained from the NCBI data repository (www.ncbi.nlm.nih.gov) were used for multiple sequence alignment using the BioEdit sequence editor (Version 7.2.5). The homology model of xynA was built using an automated Swiss-modeling server [56], and the difference in amino acid of VSBD5 and KBFB4 was visualized and analyzed using an open-source software package PyMoL version 0.97.

### In silico analysis

Physicochemical parameters such as molecular weight, theoretical pI, extinction coefficient, instability index, aliphatic index, grand average of hydrophobicity (GRAVY) were analyzed using the ExPASy-ProtParam tool (http://web.expasy.org/protparam). The signal peptide was predicted using the tool in the SignalP-5.0 server (http://www.cbs.dtu.dk/services/SignalP/) [57].

## Supplementary Information

**Additional file 1: Figure S1.** Xylanase production by thermophilic bacteria. (a) Enrichment of biomass samples in the hot springs of Himachal Pradesh to isolate thermophilic bacteria. (b) Plate screening for the xylanase activity (the enzyme activity for the bacterial isolates was visualized as a pale zone around the colony (indicated by arrow) using 1% Congo red dye. (c) The morphology of strains KBFB4 and VSDB5 on the tryptic soy agar plate. KBFB4 colonies had a granular consistency, whereas VSDB5 colonies were off-white with a creamy consistency. **Figure S2.** Secondary structure analysis of xylanase protein from *Bacillus* strains VSDB5 and KBFB4. The secondary structure indicates that the structure is composed of random coils, extended sheets, α-helices, and to a lesser extent beta-turn regions.

## Data Availability

All data generated or analyzed during this study are included in this published study. Any additional information will be provided by the corresponding author upon communicating with usiva@tnau.ac.in.

## Abbreviations

DNS: Dinitro salicylic acid
16S rRNA: 16S ribosomal DNA
KBFB4: Kalath banana fiber bacteria 4
VSDB5: Vashisht saw dust bacteria 5
BLAST: Basic Local Alignment Search Tool
GH11: Glycosyl hydrolase 11
IU mL^−^^1^: International units per milliliter
