# Supplementary material for: Glycosyl hydrolase 11 (xynA) gene with xylanase activity from thermophilic bacteria isolated from thermal springs
Source: Microb Cell Fact. 2022 Apr 15;21:62. doi: 10.1186/s12934-022-01788-3 (PMC9013152; doi:10.1186/s12934-022-01788-3)
Supplement: Supplementary file 1 — Additional file 1: Figure S1. Xylanase production by thermophilic bacteria. (a) Enrichment of biomass samples in the hot springs of Himachal Pradesh to isolate thermophilic bacteria. (b) Plate screening for the xylanase activity (the enzyme activity for the bacterial isolates was visualized as a pale zone around the colony (indicated by arrow) using 1% Congo red dye. (c) The morphology of strains KBFB4 and VSDB5 on the tryptic soy agar plate. KBFB4 colonies had a granular consistency, whereas VSDB5 colonies were off-white with a creamy consistency. Figure S2. Secondary structure analysis of xylanase protein from Bacillus strains VSDB5 and KBFB4. The secondary structure indicates that the structure is composed of random coils, extended sheets, α-helices, and to a lesser extent beta-turn regions. [file 12934_2022_1788_MOESM1_ESM.docx]

**Fig. S1. Xylanase production by thermophilic bacteria. (a)** Enrichment of biomass samples in the hot springs of Himachal Pradesh to isolate thermophilic bacteria. **(b)** Plate screening for the xylanase activity (the enzyme activity for the bacterial isolates was visualized as a pale zone around the colony (indicated by arrow) using 1% Congo red dye. **(c)** The morphology of strains KBFB4 and VSDB5 on the tryptic soy agar plate. KBFB4 colonies had a granular consistency, whereas VSDB5 colonies were off-white with a creamy consistency.


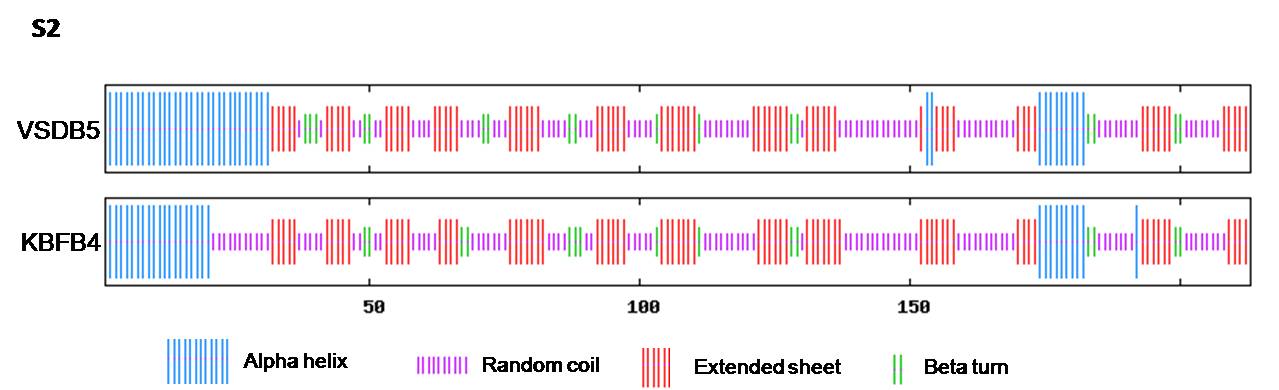


**Fig. S2. Secondary structure analysis of xylanase protein from *Bacillus* strains VSDB5 and KBFB4.** The secondary structure indicates that the structure is composed of random coils, extended sheets, α-helices, and to a lesser extent beta-turn regions.
